# The Two-Sided Effect of Leader Unethical Pro-organizational Behaviors on Subordinates’ Behaviors: A Mediated Moderation Model

**DOI:** 10.3389/fpsyg.2020.572455

**Published:** 2020-10-22

**Authors:** Peng Wen, Cheng Chen, Silu Chen, Yuyang Cao

**Affiliations:** ^1^School of Economics and Business Administration, Central China Normal University, Wuhan, China; ^2^College of Public Administration, Huazhong University of Science and Technology, Wuhan, China

**Keywords:** unethical pro-organizational behavior, Machiavellianism, organizational citizenship behavior, unethical behavior, social information processing theory

## Abstract

Research suggests that unethical pro-organizational behavior (UPB) has two conflicting characteristics: unethical and pro-organizational. However, little attention has been paid to the negative and positive outcomes of UPB. Therefore, the present study aimed to fill this gap by examining a mediated moderation model on the effects of leader UPB on their subordinates’ behaviors. Based on social information processing theory and three-wave survey data from 204 supervisor-subordinate dyads in China, we found that the mixed relationships between leader UPB and subordinates’ behaviors were dependent on the leader’s Machiavellianism. Specifically, for high Machiavellian leaders, their UPB was positively related to subordinates’ unethical behaviors *via* subordinates’ moral disengagement. For low Machiavellian leaders, their UPB was positively related to subordinates’ organizational citizenship behaviors *via* their organizational identification. The theoretical contributions and practical implications of the findings are discussed.

## Introduction

Unethical pro-organizational behavior (UPB) is defined as “actions that are intended to promote the effective functioning of the organization or its members and violate core societal values, mores, laws, or standards of proper conduct” (Umphress and Bingham, 2011, p. 622). UPB is often used by leaders and employees in order to help organizations win under fierce market competition (Umphress et al., 2010; Effelsberg and Solga, 2015). Previous research has mainly focused on the predictors of UPB, such as individual characteristics, leadership style, and organizational context (Miao et al., 2013; Effelsberg et al., 2014; Effelsberg and Solga, 2015; Graham et al., 2015; Wang and Li, 2019). However, little is known about the consequences of UPB; specifically, how leaders’ UPB affects their subordinates. Leaders are important social actors and ethical role models who influence subordinates’ attitudes and behaviors (Brown and Treviño, 2014). Examining the effect of leaders’ UPB on subordinates will promote understanding of the functions or roles of UPB in the organizational context, and provide corresponding implications for the effective management of UPB.

According to its definition, UPB has two conflicting characteristics: unethical and pro-organizational. On one hand, *unethical* means that UPB deviates from societal norms and may ultimately cause harm; conversely, *pro-organizational* means that UPB plays a positive role in benefiting or helping the wider organization (Umphress et al., 2010). One interesting question is whether leaders’ UPB would lead to subordinates’ positive and negative behaviors. In the present research, we attempt to answer this question by using social information processing theory as the overarching framework. Social information processing theory (Salancik and Pfeffer, 1978) suggests that the social information surrounding individuals provides social clues that help them to interpret the work environment. Different interpretations of the working environment will lead to different attitudes and behaviors. Considering the complexity of UPB, we infer that leaders’ UPB may provide employees with two types of opposing social information and then cause both negative behaviors (e.g., unethical behaviors) and positive behaviors [e.g., organizational citizenship behavior (OCB)].

Further, although individual UPB has the appearance of organization-serving intentions on the surface (Umphress and Bingham, 2011), few studies have discussed the real motives related to why people engage in UPB. Recent research has found that there are differences or even conflicts between the publicly (un)ethical behaviors of leaders and their privately held motives (Den Hartog and Belschak, 2012). Social information processing theory also suggests that the formation of employees’ attitudes and behaviors is not only affected by the characteristics of information itself but also by the relevant aspects of the information sender (Miller and Monge, 1985). Thus, it is necessary to examine whether leaders’ internal traits will influence subordinates’ perceptions of their leader’s public style or actions and their subsequent behaviors (Chuang and Chiu, 2018). In the present research, we chose one leader personality trait closely related to their moral decision making – namely Machiavellianism, defined as a social conduct strategy involving the manipulation of others for personal gain (Wilson et al., 1996). This paper will examine the moderating role of Machiavellianism in the relationships between leaders’ UPB and subordinates’ unethical behaviors and OCB. Further, in order to deeply understand the above process, we investigated the interactive effect of leader Machiavellianism and UPB on their subordinates’ behaviors *via* two cognitive variables: moral disengagement and organizational identification. Specifically, we examined the mediating role of moral disengagement in the interactive effect of leader Machiavellianism and UPB on subordinates’ unethical behaviors, as well as the mediating role of organizational identification in the interactive effect of leader Machiavellianism and UPB on subordinates’ OCB.

**Figure 1:**
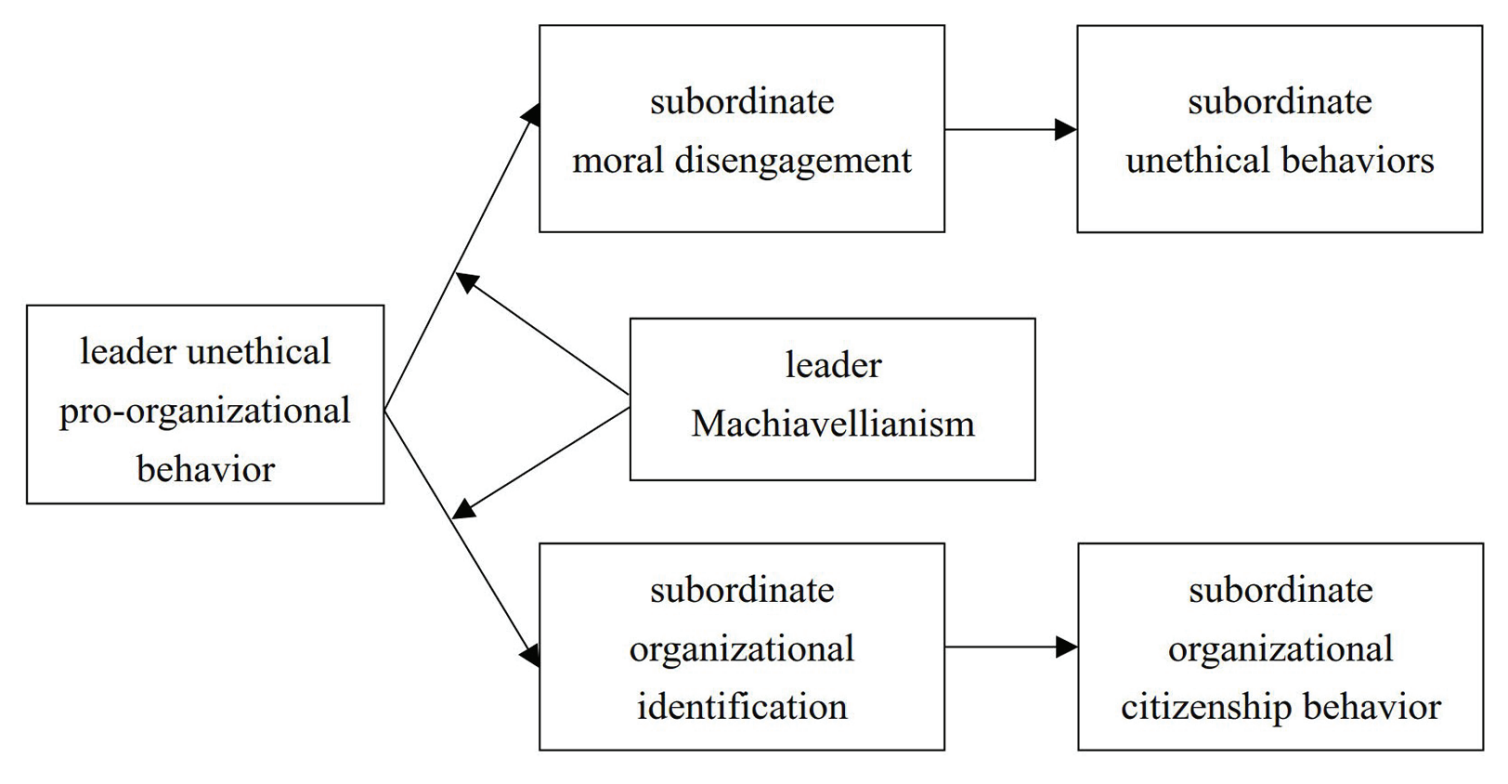
Research model.

In so doing, we make several contributions. First, this study contributes to a theoretical understanding of the mixed consequences of UPB. Previous studies have focused on the antecedent variables of UPB, but not enough attention has been paid to its possible complex results (Wang et al., 2018). Based on the supervisor-subordinate context, this study focuses on the dual effects of leaders’ UPB on subordinates’ unethical behaviors and OCB. This will provide a deeper understanding of the complex functions of UPB in an organization. Second, this study contributes to the literature on UPB by examining the moderating effect of Machiavellianism. Although the existing UPB literature has proposed the nature of its complexity, it mainly emphasizes its apparent intention and external behavior (Umphress and Bingham, 2011), and seldom deals with its true motivation. As an important personality trait related to individual moral decision making, Machiavellianism will provide important social cues for subordinates to infer the real motive behind why leaders conduct UPB. The comprehensive social information also helps subordinates make corresponding judgments and behavioral responses to leaders’ UPB. Finally, we contribute to the literature on (un)ethical leadership processes by examining the cognitive mechanism in the interactive effect of leaders’ UPB and Machiavellianism on subordinates’ behaviors. Previous research on (un)ethical leadership processes has mainly focused on social learning and social exchange perspectives (Brown et al., 2005; Qian et al., 2017). Our study adopts the social cognitive perspective and proposes that employee moral disengagement and organizational identification work as the psychological mechanism underlying the link between the interaction of leaders’ UPB and Machiavellianism and employee behaviors, which provides a novel theoretical perspective in inspecting (un)ethical leadership processes. Our research model is shown in Figure 1.

## Literature Review and Hypotheses

### Leaders’ UPB and Subordinates’ Unethical Behavior and OCB

A series of business scandals (e.g., Enron, Toshiba) have resulted in greater attention being placed on unethical behaviors in organizational settings. Generally, unethical behavior refers to “any organizational member action that violates widely accepted (societal) moral norms” (Kish-Gephart et al., 2010, p. 2), and is then treated as harmful to the sustainable development of the overall organization (Treviño et al., 2006, 2014; Carberry et al., 2018). However, recent research has found that employees may engage in a new form of unethical behavior in order to benefit their organizations, referred to as UPB (Umphress et al., 2010; Effelsberg and Solga, 2015). For example, one salesman may lie to customers to protect the company image, or one financial manager may falsify financial data in order to make the company have more positive financial data. The new concept of UPB was proposed by Umphress et al. (2010). According to their view, UPB incorporates two key components. First, UPB is unethical, meaning that the behavior violates global societal norms rather than merely violating organizational norms. The second component is pro-organizational, meaning that the behavior is intended to benefit the organizations or its members.

The two different characteristics of UPB may bring two different results. According to the theory of social information processing (Salancik and Pfeffer, 1978), the behavior of superiors is important social information for subordinates to interpret in the working environment, which leads to different behaviors of individuals. On the one hand, because the UPB implemented by leaders has the unethical attribute (Umphress et al., 2010; Umphress and Bingham, 2011), it will make the subordinates feel that the behavior violating the social norms in the organization is accepted by the organization or its leaders. This will give them greater courage to carry out various types of unethical behaviors themselves. In other words, leaders’ UPB may be positively correlated to subordinates’ unethical behavior. On the other hand, since UPB is designed to promote the development of organizations and their members, it will make subordinates pay attention to the importance of altruistic behaviors in the organization and then implement similar behaviors accordingly. At the same time, the pro-organizational side of UPB may also help leaders establish a high-quality exchange relationship with their subordinates (Wang et al., 2018). Since the superior is the agent of the organization, the subordinate may reciprocate to the superior and organization by implementing positive behaviors, such as OCB, defined as behavior that is not formally requested or directly rewarded but can be functional to the operations of an organization (Gouldner, 1960; Smith et al., 1983; Cropanzano and Mitchell, 2005). This means that leaders’ UPB may also trigger the OCB of the subordinate.

### The Moderating Role of Leader Machiavellianism

Machiavellianism is viewed as one type of stable personality trait (Wilson et al., 1996; Paulhus and Williams, 2002). In the famous novel, *The Prince*, Niccolo Machiavelli (1469–1527), an Italian politician and philosopher, suggests that people should aim to achieve political goals in the interests of others and create personas to establish generally good impressions. He, therefore, became known as the source of the notion that the ends justify the means. From this, Christie and Geis (1970) were the first to develop the construct of Machiavellianism used to describe individual tendencies to manipulate others in order to benefit oneself. They argued that Machiavellianism includes the following three dimensions: interpersonal tactics, a cynical view of human nature, and a disregard for conventional morality. There is evidence indicating that leaders with high or low Machiavellianism engage in different behaviors. High Machiavellian leaders are skilled at creating a positive impression, including crafting an overall charismatic image (Gardner and Avolio, 1995). Deluga (2001) found that presidential Machiavellianism was positively connected with charismatic leadership. Moreover, previous research indicates that leaders with high Machiavellianism are directive toward and inconsiderate of their followers (Dahling et al., 2009). In contrast, low Machiavellian leaders focus primarily on fairness, honesty, and compassion (Sendjaya et al., 2016; Shafer and Lucianetti, 2018).

We argue that a leader’s Machiavellianism positively moderates the relationship between their UPB and their subordinates’ unethical behaviors and negatively moderates the relationship between leader UPB and subordinate OCB. As stated, Machiavellian leaders have a strong self-serving tendency and desire for both achievements and social status (Kapoutsis et al., 2013). They are skilled at performing various actions, including immoral ones (e.g., manipulation and deception), in order to attain personal objectives (Sakalaki et al., 2007). The motives underlying these leaders’ UPB appear to be for the benefit of the wider organization but are actually for their own purposes, including promoting their organizational status or creating an overall positive impression. Therefore, when high Machiavellian leaders conduct UPB, subordinates tend to interpret this behavior as derived from a self-serving motivation according to social information processing theory (Salancik and Pfeffer, 1978). The difference between public UPB and real motive will lead subordinates to think that the superior is an immoral leader, which will further result in more attention to the unethical side of their leader’s UPB. Moreover, they may not worry about being punished by organizations or leaders for conducting unethical behaviors as their leaders are perceived as unethical role models (Treviño and Brown, 2005). All these factors indicate that leaders’ UPB may result in more subordinates’ unethical behaviors when leaders are high in Machiavellianism.

Recent research has provided indirect evidence for the above arguments. For example, Den Hartog and Belschak (2012) found that leader Machiavellianism negatively moderates the relationship between ethical leadership and subordinate work engagement. For high Machiavellian leaders, their ethical behaviors often draw subordinates’ attention to their own self-interest, utilitarian needs, and immoral characteristics, resulting in low subordinate work engagement. In this study, we adopted a similar view in assuming that high Machiavellian leaders’ UPB will result in their subordinates engaging in negative behaviors; in other words, actions that are unethical. Therefore, we propose the following hypothesis:

*H1*: Leader Machiavellianism positively moderates the relationship between leader UPB and their subordinates’ unethical behaviors. Specifically, the relationship is stronger for leaders with high Machiavellianism compared to those with low Machiavellianism.

Compared to high Machiavellian leaders, low Machiavellian leaders emphasize moral value, honesty, and transparency (Christie and Geis, 1970). They are unlikely to engage in unethical behaviors for their own self-interest. In fact, conducting UPB comes with a risk of moral condemnation and even legal penalties because the behavior is unethical in nature (Umphress et al., 2010). Thus, when low Machiavellian leaders conduct UPB, they might have to engage in these behaviors to promote organizational survival and development, with them then being viewed as people with motivations of self-sacrifice and devotion. In this case, subordinates have increased respect for and trust in their leaders. Therefore, these subordinates pay greater attention to the positive (pro-organizational) aspect of their leader’s UPB. Meanwhile, leaders’ UPB viewed as a signal of self-sacrifice or organization-serving motives makes employees feel that they are in a dedicated and mutually beneficial work environment (Cremer and Van Knippenberg, 2005). Subordinates will conduct more OCB with the aim of reciprocating considerate treatment from their supervisors or organizations. Conversely, subordinates often feel that high Machiavellian leaders conduct UPB based on self-serving intentions and, as such, have decreased trust in their leaders and the sense of reciprocity with leaders and organizations. In this case, it is difficult for leaders who conduct UPB to advance subordinate OCB. Therefore, we propose the following hypothesis:

*H2*: Leader Machiavellianism negatively moderates the relationship between leader UPB and subordinate OCB. Specifically, the relationship is stronger for leaders with low Machiavellianism than those with high Machiavellianism.

### The Mediating Role of Moral Disengagement

Moral disengagement is defined as a series of interrelated cognitive mechanisms that serve to deactivate the moral self-regulatory processes that are supposed to inhibit immoral or unethical behaviors (Bandura, 1999). Moral disengagement has been viewed as both a trait variable (Gini et al., 2014) and a state variable (Duffy et al., 2012; Zhang et al., 2018). The present research will focus on its aspect of moral cognition and treat it as a state variable that may be altered by leaders. An individual’s self-regulatory function plays an important role in the reduction of inconsistencies occurring when their external behaviors violate their internalized moral standards. The concept of moral disengagement indicates that it leads to a failure within this self-regulatory process. Specially, Bandura (1999) proposed that moral disengagement functions through a set of eight interrelated cognitive mechanisms. The first set of these mechanisms, including *moral justification*, *euphemistic labeling*, and *advantageous comparison*, cognitively restructures one’s unethical behaviors in order to make them appear less harmful. The second set of mechanisms uses *displacement of responsibility*, *diffusion of responsibility*, and *distortion of consequences* in order to obscure or distort the impacts of one’s harmful actions. The final two mechanisms, *dehumanization* and *attribution of blame*, reduce an individual’s identification with the recipients of their harmful behaviors.

We propose that high Machiavellian leaders’ UPB will lead to subordinates’ moral disengagement for two reasons. First, subordinates may have moral disengagement through learning from high Machiavellian leaders’ UPB. As leaders are often viewed as role models of subordinates, subordinates may believe that leaders’ behaviors are acceptable and reasonable and learn from those behaviors. As stated, subordinates will pay attention to the self-serving intentions of high Machiavellian leaders engaging in UPB and tend to treat the leaders as immoral persons. Then, they will develop a moral cognitive bias that unethical behaviors are normal (Ashforth and Anand, 2003; Valle et al., 2019). In this case, high moral disengagement of subordinates appears. Second, subordinates are prone to find excuses in order to justify unethical behaviors through the diffusing of their own responsibility. Because leaders often have the legal rights to reward and punish their subordinates, subordinates will choose to follow and obey the leaders to avoid being punished (Podsakoff et al., 2006). As mentioned above, high Machiavellian leaders’ UPB tend to make subordinates focus on its unethical side. As a result, employees will consider it a wise choice to perform unethical behaviors. Even if these behaviors have negative effects on organizations, they may argue that they were obeying the orders of their leaders, so they should not be held responsible for their unethical actions; this responsibility should be taken by the leaders who command them (Zimbardo, 1995).

Moral disengagement deactivates the moral self-regulation processes and lead individuals to fail to act on their moral standards (Bandura, 1986). When individuals exercise moral disengagement, they do not feel guilt, regret, or remorse after they have conducted unethical behaviors violating their own moral standards (Bandura, 1999); they try to defend their unethical behaviors by making them appear to be morally acceptable. In this case, we believe that the presence of high moral disengagement will result in unethical behaviors. A great deal of research provides evidence that individual moral disengagement leads to unethical behaviors through the relief of feelings of guilt and self-censure (Bandura et al., 1996; Aquino et al., 2007; Detert et al., 2008; Huang et al., 2017). Moore et al. (2012) proposed that when compared to other individual traits or cognitive variables related to (un)ethical decisions (e.g., moral identity and moral reasoning), moral disengagement remains the best predictor of unethical behaviors.

Taken together, high Machiavellian leaders’ UPB will lead subordinates to learn from and obey leaders. Then, subordinates will have a moral cognitive bias toward unethical behaviors, which is moral disengagement. The cognition treating unethical behaviors as morally acceptable will result in more unethical behaviors. Therefore, we propose the following hypothesis:

*H3*: The interactive effect of leader Machiavellianism and UPB on subordinate unethical behaviors is mediated by subordinate moral disengagement.

### The Mediating Role of Organizational Identification

Organizational identification is defined as the perception of oneness with or belongingness to an organization or institution (Mael and Ashforth, 1992; Smidts et al., 2001; Van Knippenberg et al., 2002). According to this definition, organizational identification is seen as a perceptual or cognitive construct that reflects the relationship between employees’ self-concept and the organization itself (Mael and Ashforth, 1992). As mentioned, subordinates will respect and trust low Machiavellian leaders who conduct UPB. As leaders are usually treated as the agents of an organization, the trust in them is then transferred to the organization itself (Hui et al., 2004). Scholars (e.g., Tyler and Blader, 2000; Van Knippenberg et al., 2004) suggest that people identify more with organizations when trust is present. Furthermore, as low Machiavellian leaders are considered to conduct UPB with the motivations of self-sacrifice and collective benefits, subordinates’ pride and happiness tend to be enhanced. In this case, subordinates will recognize their own identity and accept organizational missions or values so as to increase the sense of organizational identity (Meleady and Crisp, 2017). Recent empirical research has found that self-sacrificial leadership can promote subordinates’ organizational identification (e.g, Li et al., 2016). This suggests that low Machiavellianism leaders’ UPB may link subordinates’ identities to the collective identity of their organizations, resulting in high organizational identification.

Organizational identification results in high organizational dependence, recognition, and loyalty. Organizational identification elicits a sense of oneness with the organization, resulting in employees promoting positive responses toward their employing organization and treating the organization’s goals as their own (Van Knippenberg, 2000; Ellemers et al., 2004). In turn, employees have increased motivation to help their organizations through conducting beneficial behaviors, such as OCB (Wu et al., 2016). Supporting these arguments, previous research has indicated that organizational identification is positively related to individual behaviors such as OCB (e.g., Pratt et al., 2006; Walumbwa et al., 2009). Meanwhile, in a meta-analysis, Riketta (2005) also found that overall measures of organizational identification were positively correlated with extra-role behaviors (*r* = 0.35, *p* < 0.001). Thus, when subordinates have high organizational identification for the UPB conducted by leaders with low Machiavellianism, they then become motivated to conduct OCB in order to repay leaders for considering their interests and help the organization attain its goals.

Taken together, low Machiavellianism leaders’ UPB will increase subordinates’ trust and respect for leaders, and their pride and happiness in the organization. These will lead to high organizational identification, which in turn will promote positive responses toward their organization by conducting more OCB. Therefore, we propose the following hypothesis:

*H4*: The interactive effect of leader Machiavellianism and UPB on subordinate OCB is mediated by subordinate organizational identification.

## Materials and Methods

### Participants and Procedure

Data were collected from full-time employees and their leaders from a large-scale financial organization in China. Matching questionnaires were distributed to leaders and subordinates, with each leader rating two or three of their subordinates. Surveys were administered across three time periods. The time lag between each wave of the survey was 4 weeks. In the time 1 survey, leaders were asked to rate their own Machiavellianism and the extent to which they engaged in UPB. Then, subordinates reported their organizational identification, moral disengagement, and demographic characteristics in the time 2 survey. Finally, at the time 3 point, the leaders assessed the OCB of their subordinates who responded at time 2, with these subordinates also reporting on their own unethical behaviors. We provided paper-pencil survey packages and asked participants to return the questionnaires, using a post-paid envelope, to us. Only completely filled-out, matched supervisor-subordinate questionnaires were included in the final analyses. The sample consisted of 204 completed supervisor-subordinate dyads, resulting in a response rate of 55.3%.

Regarding employees’ demographics, the sample was 51% male. The age of most participants (79.4%) was below 35 years. About two-thirds of the participants (62.2%) were educated to at least college-level. Most participants had been employed with the company for 1–5 years (62.7%). Detailed demographic characteristics of employees are reported in Table 1.

**Table 1:** Demographic characteristics of participants (*N* = 204).

Variables	Type	Number	Percentage		Variables	Number	Percentage
Age	18–25 years	46	22.5%	Education	Middle school	33	16.2%
	26–30 years	84	41.2%		High school/technical school diploma	44	21.6%
	31–35 years	32	15.7%		Associate degree	36	17.6%
	36–40 years	25	12.3%		Bachelor	80	39.2%
	41–50 years	13	6.3%		Master and above	11	5.4%
	over 50 years	4	2.0%	Organizational tenure	Below 1 year	8	3.9%
Gender	Male	104	51%		1–2 years	54	26.5%
					3–5 years	74	36.2%
	Female	100	49%		6–10 years	55	27%
					Above 10 years	13	6.4%

### Measures

Because this study was conducted in China but all scales used were originally written in English, translation and back-translation were conducted in a manner consistent with established cross-cultural translation procedures (Brislin, 1980). All items in the questionnaire, except for the demographic variable, were measured using a 5-point Likert scale (1 = strongly disagree and 5 = strongly agree).

#### Unethical Pro-organizational Behavior

We used the 6-item scale developed by Umphress et al. (2010) to measure leader UPB. Each leader was asked to report the extent to which he/she engage in the following behaviors. Example items are “if it would help our organization, I would misrepresent the truth to make our organization look good” and “if needed, I would conceal information from the public that could be damaging to our organization” (Cronbach’s *α* = 0.91).

#### Machiavellianism

We asked leaders to rate their degree of Machiavellianism with the short 8-item version of Mach-IV scale of Christie and Geis (1970). Example items are “it is wise to flatter important people” and “never tell anyone the real reason you did something unless it is useful to do so” (Cronbach’s *α* = 0.93).

#### Organizational Identification

Subordinates completed the 6-item scale of Mael and Ashforth (1992). Sample items are “this company’s successes are my successes” and “when someone criticizes my company, it feels like a personal insult” (Cronbach’s *α* = 0.89).

#### Moral Disengagement

Moral disengagement was measured with 8-item scale of Moore et al. (2012). Sample items are “considering the ways people grossly misrepresent themselves, it is hardly a sin to inflate your own credentials a bit” and “people should not be held accountable for doing questionable things when they were just doing what an authority figure told them to do” (Cronbach’s *α* = 0.93).

#### Organizational Citizenship Behavior

Using the 6-item OCB-Organization scale developed by Williams and Anderson (1991), leaders reported subordinate OCB. Sample items are “attendance at work is above the norm” and “complains about insignificant things at work (R)” (Cronbach’s *α* = 0.87).

#### Unethical Behaviors

Subordinates’ self-report unethical behaviors were measured with Peterson’s (2002) 9-item scale of unethical behavior. Sample items are “calling in sick to take a day off even though other employees will have to make up for the slack” and “give gifts/favors in exchange for preferential treatment” (Cronbach’s *α* = 0.92).

#### Control Variables

Following previous research (Roxas and Stoneback, 2004; Lam et al., 2015; Ni and Li, 2017), four subordinates’ demographic characteristics variables were measured and included in the regression analysis: age (1 = 18–25 years, 2 = 26–30 years, 3 = 31–35 years, 4 = 36–40 years, 5 = 41–50 years, and 6 = over 50 years), education level (1 = middle school, 2 = high school/technical school diploma, 3 = associate degree, 4 = bachelor, and 5 = master and above), gender (1 = male and 0 = female), and organizational tenure (1 = below 1 year, 2 = 1–2 years, 3 = 3–5 years, 4 = 6–10 years, and 5 = above 10 years).

## Results

### Descriptive Statistics

The means, standard deviations, and intercorrelations among variables are presented in Table 2. The results indicate that leader UPB is positively related to subordinate unethical behaviors (*r* = 0.29, *p* < 0.001) but not related to OCB (*r* = 0.08, n.s.). Moreover, leader UPB is positively related to subordinate organizational identification (*r* = 0.18, *p* < 0.05) and moral disengagement (*r* = 0.25, *p* < 0.001).

**Table 2:** Descriptive statistics.

Variables	Mean	*SD*	1	2	3	4	5	6	7	8	9
1. Leader UPB	2.78	0.92									
2. Leader Machiavellianism	3.40	0.90	−0.17^*^								
3. Subordinate organizational identification	3.79	0.87	0.18^*^	−0.07							
4. Subordinate moral disengagement	3.21	0.95	0.25^***^	−0.10	0.07						
5. Subordinate OCB	3.58	0.86	0.08	−0.05	0.21^**^	0.01					
6. Subordinate unethical behaviors	2.00	0.80	0.29^***^	0.05	0.07	0.26^***^	−0.15^*^				
7. Age	2.45	1.25	−0.06	0.02	−0.06	−0.18^**^	0.12	−0.10			
8. Education	2.96	1.22	−0.02	0.06	0.08	−0.08	0.02	−0.12	−0.01		
9. Gender	0.51	0.50	−0.06	0.07	0.00	−0.14^*^	−0.03	0.06	−0.07	0.15^*^	
10. Organizational tenure	3.05	0.97	0.09	0.01	0.03	0.03	0.09	0.02	−0.01	−0.32^***^	−0.08

* *p* < 0.05;

** *p* < 0.01;

*** *p* < 0.001.

### Hypotheses Tests

#### Confirmatory Factor Analyses

We used confirmatory factor analysis (CFA) to examine the distinction validity of the main variables (leader UPB and Machiavellianism, and subordinate organizational identification, moral disengagement, OCB, and unethical behaviors). The results in Table 3 show, six-factor model fitted the data well (*χ*^2^ = 1307.91, *df* = 845, RMSEA = 0.05, CFI = 0.95, NFI = 0.89), whereas other models exhibited significantly poorer fit. These results mean that there is good distinction validity among the main variables in the present study.

**Table 3:** Measure model comparison.

Models	*χ*^2^	*df*	▵*χ*^2^	RMSEA	CFI	NFI
Six-factor model (baseline model): UPB; Mac; OI; MD; OCB; and UB	1307.91	845		0.05	0.95	0.89
Five-factor model: MD and UB were combined into one factor; UPB; Mac; OI; and OCB	3566.71	850	451.76^***^	0.13	0.86	0.81
Five-factor model: OI and OCB were combined into one factor; UPB; Mac; MD; and UB	2130.62	850	164.54^***^	0.09	0.91	0.85
Three-factor model: UPB, MD, and UB were combined into one factor; OI and OCB were combined into one factor; and Mac	5551.67	857	353.65^***^	0.16	0.76	0.71
Two-factor model: UPB, MD, UB, OI, and OCB were combined into one factor and Mac	6763.3	859	389.67^***^	0.18	0.7	0.65
One factor model: Six factors were combined into one factor	9153.58	860	523.04^***^	0.22	0.6	0.45

UPB, leader unethical pro-organizational behavior; Mac, leader Machiavellianism; OI, organizational identification; MD, moral disengagement; OCB, organizational citizenship behavior; UB, unethical behaviors.

*** *p* < 0.001.

#### Regression Analysis

Because most leaders evaluated the OCB of more than one subordinate, we conducted within-and-between analysis (WABA) to test the independence of the variable (Yammarino and Markham, 1992). The result (*F* = 1.25, *p* > 0.05) showed that there were no systematic differences in supervisors’ ratings of OCB. Therefore, we used hierarchical regression to examine proposed hypotheses at individual level. All interaction variables were mean-centered to reduce multicollinearity and enhance the interpretability of the interactions (Aiken et al., 1991).

Hypothesis 1 stated that leader Machiavellianism positively moderated the relationship between leader UPB and subordinate unethical behaviors. The results in Table 4 showed that, after controlling the effect of other variables on unethical behaviors, the beta coefficient for the interaction term (leader UPB by leader Machiavellianism) was statistically significant (*β* = 0.17, *p* < 0.05, ▵*R*^2^ = 0.03, Model 4). Thus, Hypothesis 1 was supported. The results in Table 3 also supported Hypothesis 2 that leader Machiavellianism negatively moderated the relationship between leader UPB and subordinate OCB (*β* = −0.25, *p* < 0.01, ▵*R*^2^ = 0.06, Model 9).

**Table 4:** Hierarchical regression analysis.

Variables	Subordinate moral disengagement		Subordinate unethical behaviors			Subordinate organizational identification		Subordinate OCB		
	M1	M2	M3	M4	M5	M6	M7	M8	M9	M10
Age	−0.17^*^	−0.16^*^	−0.08	−0.07	−0.03	−0.05	−0.06	0.12	0.11	0.12
Education	−0.06	−0.04	−0.15^*^	−0.13	−0.11	0.09	0.07	0.06	0.03	0.00
Gender	−0.13	−0.12	0.08	0.09	0.11	0.00	−0.01	−0.02	−0.03	−0.01
Organizational tenure	−0.03	−0.03	−0.06	−0.06	−0.05	0.04	0.04	0.10	0.10	0.10
Leader UPB	0.22^**^	0.21^**^	0.31^***^	0.30^***^	0.28^***^	0.17^*^	0.18^*^	0.08	0.09	0.03
Leader Mac	−0.05	−0.08	0.11	0.07	0.09	−0.05	−0.01	−0.04	0.02	0.02
Subordinate organizational identification					0.06					0.16^*^
Subordinate moral disengagement					0.18^*^					0.06
leader UPB × leader Mac		0.15^*^		0.17^*^	0.14		−0.16^*^		−0.25^**^	−0.18^*^
Subordinate organizational identification × leader Mac					0.10					0.14^*^
Subordinate moral disengagement × leader Mac					−0.03					−0.05
▵*R*^2^	0.11	0.02	0.12	0.03	0.04	0.05	0.02	0.03	0.06	0.05
▵*F*	4.17^**^	4.54^*^	4.66^***^	5.88^*^	2.40^+^	1.54	4.95^*^	1.09	11.92^**^	2.64^*^

Mac, Machiavellianism.

+ *p* < 0.10;

* *p* < 0.05;

** *p* < 0.01;

*** *p* < 0.001.

**Figure 2:**
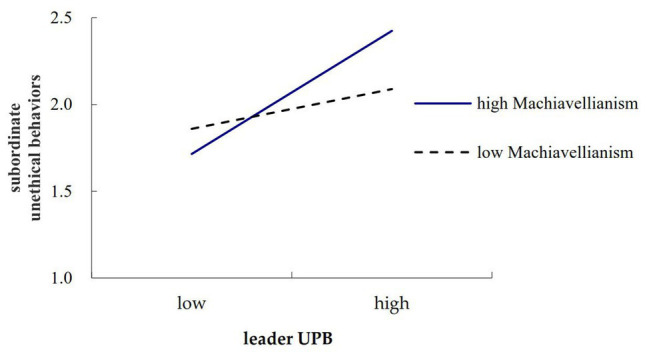
Moderating effect of leader Machiavellianism on the relationship between leader unethical pro-organizational behavior (UPB) and subordinate unethical behaviors.

**Figure 3:**
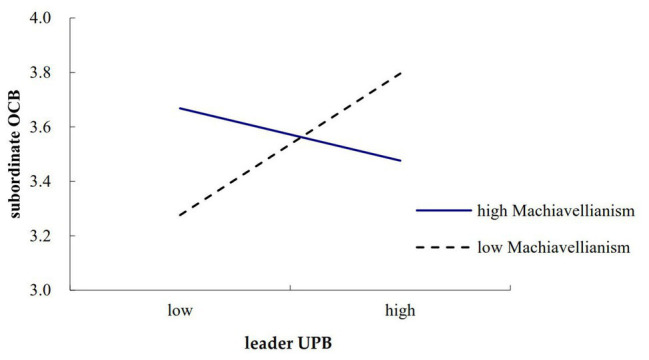
Moderating effect of leader Machiavellianism on the relationship between leader UPB and subordinate organizational citizenship behavior (OCB).

To further clarify the moderated effect of leader Machiavellianism, we examined separate simple slopes depicting the relationship between leader UPB and subordinate unethical behavior, and the relationship between leader UPB and subordinate OCB. In Figures 2, 3, separate plots were drawn for individuals whose scores on the moderator were one standard deviation below the mean and one standard deviation above the mean (Aiken et al., 1991). Results showed that the relationship between leader UPB and subordinate unethical behaviors was strengthened when leader Machiavellianism was high (*β* = 0.39, *p* < 0.001) rather than low (*β* = 0.12, n.s.). Similarly, results showed that the relationship between leader UPB and subordinate OCB was strengthened when leader Machiavellianism was low (*β* = 0.28, *p* < 0.01) rather than high (*β* = −0.11, n.s.). These results further verified Hypotheses 1 and 2.

Hypothesis 3 proposed that subordinate moral disengagement mediated the interactive effect of leader Machiavellianism and UPB on subordinate unethical behavior. The mediated moderation was tested based on the procedures outlined by Muller et al. (2005). A mediated moderation effect is supported if four conditions are met: (1) the interaction between the independent variable and the moderator is significantly related to the mediator; (2) after controlling for other predictors, the interaction is also significantly related to the dependent variable; (3) after controlling for the mediator × moderator term and other predictors, the mediator remains significantly related to the dependent variable; and (4) after controlling for the mediator, the effect of the interaction between the independent variable and the moderator on the dependent variable becomes weaker or non-significant.

Supporting Hypothesis 3, the results in Table 4 indicated that: (1) leader UPB by Machiavellianism is significantly related to subordinate moral disengagement (*β* = 0.15, *p* < 0.05, Model 2); (2) it is also significantly related to unethical behaviors (*β* = 0.17, *p* < 0.05, Model 4); (3) after controlling for the mediator × moderator term and other predictors, moral disengagement remains significantly related to unethical behavior (*β* = 0.18, *p* < 0.05, Model 5); and (4) after controlling for the mediator, the effect of the interaction between the leader UPB and leader Machiavellianism on the unethical behaviors becomes non-significant (*β* = 0.14, n.s., Model 5).

Furthermore, the results of the bootstrapping tests (Hayes, 2017) showed a positive indirect relationship between the leader UPB by Machiavellianism and subordinate unethical behaviors *via* moral disengagement [*N* = 1,000, indirect effect = 0.02, 95% biased-corrected bootstrap CI was (0.004, 0.085)]. Overall, Hypothesis 3 was supported.

We also used the same procedures to test Hypothesis 4 that subordinate organizational identification mediated the interactive effect of leader Machiavellianism and UPB on subordinate OCB. Supporting Hypothesis 4, the results in Table 4 indicated that: (1) leader UPB by Machiavellianism is significantly related to subordinate organizational identification (*β* = −0.16, *p* < 0.05, Model 7); (2) it is also significantly related to subordinate OCB (*β* = −0.25, *p* < 0.01, Model 9); (3) after controlling for the mediator × moderator term and other predictors, subordinate organizational identification remains significantly related to OCB (*β* = 0.16, *p* < 0.05, Model 10); and (4) after controlling for the mediator, the effect of the interaction between the leader UPB and leader Machiavellianism on the subordinate OCB becomes weaker (*β* = −0.18, *p* < 0.05, Model 10).

Furthermore, the results of the bootstrapping tests (Hayes, 2017) showed a negative indirect relationship between the leader UPB by Machiavellianism and subordinate OCB *via* subordinate organizational identification [*N* = 1,000, indirect effect = −0.02, 90% biased-corrected bootstrap CI was (−0.068, −0.002)]. Overall, Hypothesis 4 was supported.

#### Additional Analyses

We employed Edwards and Lambert (2007) general path analytic framework to test whether leader Machiavellianism moderates the mediating effect of subordinate moral disengagement on the relationship between leader UPB and subordinate unethical behaviors, as well as the mediating effect of subordinate organizational identification on the relationship between leader UPB and subordinate OCB. As shown in Table 5, the indirect effect of leader UPB on subordinate unethical behaviors *via* subordinate moral disengagement was not significant for both low and high leader Machiavellianism (*β* = 0.02, n.s.; *β* = 0.05, n.s.). Overall, the difference in the indirect effect was not significant (*β* = 0.03, n.s.). This suggests that leader Machiavellianism does not moderate the mediating effect of subordinate moral disengagement on the relationship between leader UPB and subordinate unethical behaviors.

**Table 5:** Moderated mediation effect analysis.

Moderator variable: Mac	Leader UPB(X) → Subordinate moral disengagement (M1) → Subordinate unethical behaviors (Y1)				
	Stage		Effect		
	First: P_M1X_	Second: P_Y1M1_	Direct: P_Y1X_	Indirect: P_M1X_ × P_Y1M1_	Total: P_Y1X_ + P_M1X_ × P_Y1M1_
Low Mac (mean − 1 s.d.)	0.08	0.22^*^	0.14	0.02	0.16
High Mac (means + 1 s.d.)	0.35^***^	0.15^*^	0.40^***^	0.05	0.45^***^
Difference	0.27^*^	−0.07	0.26^*^	0.03	0.29^*^
Moderator variable: Mac	Leader UPB(X) → Subordinate organizational identification (M2) → Subordinate OCB (Y2)				
	Stage		Effect		
	First: P_M2X_	Second: P_Y2M2_	Direct: P_Y2X_	Indirect: P_M2X_ × P_Y2M2_	Total: P_Y2X_ + P_M2X_ × P_Y2M2_
Low Mac (mean − 1 s.d.)	0.32^***^	0.28^**^	0.21^*^	0.09	0.30^*^
High Mac (means + 1 s.d.)	0.05	0.04	−0.14	0.00	−0.14
Difference	−0.27^*^	−0.24^*^	−0.35^**^	−0.09	−0.44^**^

Mac, leader Machiavellianism.

* *p* < 0.05.

** *p* < 0.01;

*** *p* < 0.001.

Moreover, Table 5 shows that the indirect effect of leader UPB on subordinate OCB *via* subordinate organizational identification was not significant for both low and high leader Machiavellianism (*β* = 0.09, n.s.; *β* = 0.00, n.s.). The difference in the indirect effect was not significant (*β* = −0.09, n.s.). Thus, the mediation effect of subordinate organizational identification on the relationship between leader UPB and subordinate OCB is not significantly altered by leader Machiavellianism.

## Discussion

In this research, we adopted a three-wave data collection strategy to test a mediated-moderation model in the two-sided effect of leaders’ UPB on their subordinates’ behaviors. Results indicate that the UPB conducted by high Machiavellian leaders positively influences their subordinates’ unethical behaviors *via* moral disengagement. Moreover, UPB conducted by low Machiavellian leaders positively influences subordinates’ OCB through the mediating role of organizational identification. Theoretical contributions, practical implications, limitations, and future research are discussed in the following sections.

### Theoretical Contributions

First, we contribute to research on UPB by empirically investigating the mixed consequences of leader UPB on the supervisor-subordinate relationship. Although there is increasing research examining the predictors of UPB (Umphress et al., 2010; Miao et al., 2013; Effelsberg et al., 2014; Yang et al., 2020), and a few studies have referred to the negative effect of UPB on the external public and stakeholders (Umphress and Bingham, 2011), we still know relatively little about its possibly mixed consequences and its functions in the organizational context. When the concept of UPB was first proposed, Umphress and Bingham (2011) called on researchers to further test its inherent complexities and explore its potential consequences in future studies. Our research is one such response to their appeal. As far as we know, we took the first step to empirically examine the two-sided consequences of UPB. Although the direct relationships between leader UPB and their subordinates’ behaviors are not the focus of this research, the regression results indicate that leader UPB is significantly and positively related to subordinates’ unethical behaviors, whereas it is not significantly related to OCB. This means that leaders’ behaviors that are focused on fostering organizational success at the cost of external stakeholders increase subordinates’ tendencies to engage in unethical behaviors harmful to their organizations. These results confirm the general assumptions about the detrimental effect of UPB on organizations, even though those engaging in it expect UPB to benefit their organization (Umphress et al., 2010).

Second, the present study contributes to research on UPB by examining the moderating role of Machiavellianism in the relationship between leaders’ UPB and subordinates’ behaviors (including their unethical behaviors and OCB). Previous research on UPB has mainly emphasized its apparent intention and external behavior (Umphress and Bingham, 2011), but less attention is paid to the real motivation behind the behavior. Through considering the moderating role of leader Machiavellianism, subordinates are able to gain more comprehensive social information in order to analyze their work context and then engage in appropriate behaviors. High Machiavellian leaders tend to treat UPB as a method for impression management and self-interest gratification. In this case, subordinates may treat leaders as unethical persons and pay close attention to the unethical side of leaders’ UPB, resulting in a high level of subordinates’ unethical behaviors. In contrast, low Machiavellian leaders are more likely to conduct UPB based on self-sacrificing and organization-serving motives. Subordinates, accordingly, emphasize the pro-organization aspect of their leaders’ UPB and tend to engage in more OCB to repay leaders for considering their interests. Our findings are consistent with previous views that there are differences or even conflicts between the public behaviors of leaders and their privately held motives (Bass and Steidlmeier, 1999; Den Hartog and Belschak, 2012). Moreover, similar to the findings of previous research (Den Hartog and Belschak, 2012; Chuang and Chiu, 2018), this study found that a leader’s individual characteristics, related to their ethical decision making, can influence their subordinates’ behaviors based on those leaders’ (un)ethical behaviors. On the whole, the investigation of the moderating role of leader Machiavellianism helps us to better understand the motivation behind UPB and corresponding behavioral responses of subordinates.

Finally, our research contributes to the literature on (un)ethical leadership processes by revealing the cognitive mechanism underlying the interactive effect of leaders’ UPB and Machiavellianism on subordinates’ behaviors. Although social learning and social exchange perspectives are widely used in the literature on (un)ethical leadership processes (Brown et al., 2005; Qian et al., 2017), these studies do not accurately describe the cognitive changes of individuals in the face of (un)ethical leadership. In recent years, increasing evidence has shown that individual cognitive processing and reasoning may play an important role in the field of behavioral ethics (Moore et al., 2012; Kennedy et al., 2017). Our research found that two important cognitive variables, namely moral disengagement and organizational identification, respectively, mediate the interactive effect of leader UPB and Machiavellianism on subordinates’ unethical behaviors and OCB. Thus, this research responds to a scholars’ call that more attention should be paid to subordinate cognitive reactions when considering the interactive effect of leaders’ public (un)ethical behaviors and their private identity (Machiavellian personality; Den Hartog and Belschak, 2012). Future research should attempt to examine the role of subordinates’ affective reactions – another important psychological process – in the above relationships.

### Managerial Implications

The present research also offers several managerial implications for organizations. First, it is very necessary for organizations to reduce and even prohibit leaders’ UPB as we found a positive relationship between leaders’ UPB and subordinates’ unethical behaviors. Multiple training methods (e.g., ethical dilemma exercises and online learning) should be used to raise leaders’ moral consciousness related to ethical decisions. Meanwhile, organizations should treat (un)ethical behaviors and decisions as important parts of performance appraisal and set high moral standards for leaders (Brown and Treviño, 2014).

Second, organizations need to remain especially vigilant about high Machiavellian leaders who conduct UPB as they will lead to more subordinates’ unethical behaviors. In contrast, low Machiavellian leaders may establish a positive image that results in subordinates selectively making positive choices in response to their UPB. Thus, when organizations hire or promote leadership candidates, some effective methods, such as scale measurement and behavior observation, should be adopted to judge their levels of Machiavellianism. Low Machiavellianism candidates should be the priority, especially in managerial positions (e.g., financial management and sales) that are often confronted with moral dilemmas.

Third, considering the mediating roles of the two cognitive variables, namely moral disengagement and organizational identification, it is suggested that organizations should take measures to increase the level of employee organizational identification that causes increased OCB and decreased moral disengagement, resulting in fewer unethical behaviors. For example, previous research has found that organizational ethical culture can effectively alter employees’ ethical cognitions that result in positive and ethical outcomes such as external whistleblowing (Kaptein, 2011). Thus, organizations should attempt to take measures to establish ethical culture. According to Schein’s (2010) research on organizational culture, organizations can set specific strategies or goals linked with ethical behaviors and provide visual organizational processes of ethical decision making through setting ethical slogans and codes of conduct.

### Limitations and Future Research

This study is not without limitations. The first limitation of our research is its use of self-report questionnaires to measure leaders’ UPB and subordinates’ unethical behaviors, which raises the possibility of a social desirability bias. However, we considered the self-report measure appropriate because it is exceedingly difficult for others to accurately report the destructive work behaviors (e.g., UPB and unethical behaviors) of the focal individuals (Umphress et al., 2010). Further, a meta-analysis comparing self-reports to other-reports assessing another sensitive behavior (counterproductive work behaviors) indicated that self-report data are more accurate compared to data collected from other-reports (Berry et al., 2012).

Second, this research only considered behavioral outcomes (e.g., OCB and unethical behaviors) as employees’ responses to their leaders’ UPB. Previous research has found that there is a slight positive relationship between leaders’ willingness to engage in UPB and follower-perceived transformational leadership (Effelsberg and Solga, 2015). More responses to leader UPB should be examined in future research. For example, are there subordinate emotional reactions (e.g., positive and negative emotions) to leader UPB? Moreover, when considering the complexity of UPB, examining how middle-level managers who engage in UPB are rated by their leaders, top-level managers, or the CEO becomes an important topic. Will they be promoted because of the benefits of UPB or be criticized due to its unethical nature?

A final limitation is related to the cultural context wherein the research was conducted. Our research was conducted in a specific context, namely that of China. Generally speaking, Chinese employees have a high power distance orientation (Brockner et al., 2001), which strengthens subordinates’ responses to their leaders’ behaviors (e.g., UPB). Thus, one must be cautious in extending our results to other societies with lower power distance. Further research should examine the relationships that our research tested in different cultural contexts.

## Data Availability Statement

The raw data supporting the conclusions of this article will be made available by the authors, without undue reservation.

## Ethics Statement

The studies involving human participants were reviewed and approved by Central China Normal University’s ethics committee. The patients/participants provided their written informed consent to participate in this study.

## Author Contributions

CC designed the study, performed the data analysis, and wrote the draft of the manuscript. PW and SC revised the manuscript. YC collected the data. All authors contributed to the article and approved the submitted version.

### Conflict of Interest

The authors declare that the research was conducted in the absence of any commercial or financial relationships that could be construed as a potential conflict of interest.
